# Trait ecology of startup plants

**DOI:** 10.1111/nph.18193

**Published:** 2022-05-24

**Authors:** Mark Westoby, Julian Schrader, Daniel Falster

**Affiliations:** ^1^ School of Natural Sciences Macquarie University Sydney NSW 2109 Australia; ^2^ Department of Biodiversity, Macroecology and Biogeography University of Goettingen Goettingen 37073 Germany; ^3^ Evolution & Ecology Research University of New South Wales Sydney NSW 2052 Australia

**Keywords:** epicormic growth, plant ecological strategies, regeneration strategy, resprout, sapling, seedling, startup, trait ecology

## Abstract

Startup plants include seedlings and basal and epicormic resprouts. It has long been held that startups have different strategies from adult plants, but theory for what trait differences to expect is limited and not yet quantitatively tested. Three applicable concepts are analogous to human startup firms, R‐shift, and trait‐growth theory. All three suggest startups should be built with lower construction costs than established plants. This appears to be almost always true in terms of leaf mass per area (LMA), though many comparisons are complicated by the startups growing in lower light. Trait‐growth theory predicts LMA should increase progressively with height or total leaf area, driven by higher conductive‐pathway costs associated with each unit leaf area, and by greater reward from slowing leaf turnover. Basal resprouts often have somewhat higher LMA than seedlings, but possibly this is simply because they are larger. A number of eminently testable questions are identified. Prospects are good for a theoretically cogent and field‐tested body of knowledge about plant startups.

## Introduction: three formulations about startups

Startup businesses are a recognized category, different in several ways from established enterprises (Box 1; Cohan, 2019; Eisenmann, 2021). Similarly, startup plants are different from established plants. Seedlings are startups, and so also are epicormic and lignotuber and root resprouts after fire and after drought, and regrowth from lower buds after a leader is damaged by drought or breakage or insects or pruning. Startup ecology is important for plant population dynamics generally, and for response to disturbances such as fire and drought.

Box 1 **Startup firms**

Startup firms are a recognized category within human economics, business management and entrepreneurship. Features that they share with startup plants include the following (Cohan, 2019; Eisenmann, 2021). (1) Large majorities fail. (2) Because they usually have small cash reserves, early and rapid returns are important. (3) Startups grow progressively into mature and stable firms; there is not a binary demarcation between startups and established businesses.

Markets and profitability are very uncertain for startup firms. This is because their products are typically novel. So‐called ‘angel’ investors accordingly spread their investment across many startups, accepting the high risk of failure in exchange for high returns from the minority of successes. Seedlings similarly experience much uncertainty, not because they are a novel product but due to the chances of finding themselves in different microsites. The genes shared by seedlings from an individual mother are analogous to an angel investor inasmuch as their success can be thought of as an aggregate across all the mother’s seed production.

The unpredictable microsites faced by individual seedlings (but not so much by resprouts) favour plastic adjustment of traits (e.g. Havrilla *et al*., 2021). In business, the ‘lean startup’ concept (Ries, 2011) advocates rapid adjustment of business plans as the situation evolves, in contrast to the traditional virtue of designing a sound plan from the outset. For both plants and firms, adjustment needs to build on investments already made, and this must restrict plasticity. The analogy is not exact, however. Lean startups experimentally cycle through multiple plans and change direction as the results indicate, whereas seedlings are plastic in response to environmental cues such as light and soil water that reflect the same challenges faced by their ancestors.

Startup viability is assessed through ‘unit economics’, where the units are customers, the sources of revenue. Key quantities for each customer are revenue per time and the duration of the customer’s relationship to the firm, which together give lifetime value, and the cost to acquire the customer in the first place. A baseline requirement is for lifetime value to exceed cost to acquire. The faster cost to acquire can be paid back, the better. These same formulations have been used in leaf economics (e.g. Chabot & Hicks, 1982; Kikuzawa, 1995; Westoby *et al*., 2002), where key elements are the dry mass cost of a unit of leaf area (leaf dry mass per area, LMA), leaf lifespan, returns per unit time (net of maintenance costs), and payback time on investment.

The idea that regeneration strategy is different from strategy for adult plants is by no means new (e.g. Grubb, 1977; Grime, 1979; Larson & Funk, 2016). Our question here is how this may translate into quantitative traits, which are widely used as indicators of species strategy (e.g. Westoby & Wright, 2006; Kunstler *et al*., 2016). Species traits have most often been measured on adults. For example, the trait methods handbook (Pérez‐Harguindeguy *et al*., 2013) advises ‘For robust comparisons across species, traits should be generally measured on reproductively mature, healthy‐looking individuals’.

Ecology often thinks of plants in the same way as economics thinks of firms, asking what trait values would maximize growth or the surplus of revenue over costs in different environmental or competitive settings (e.g. Bloom *et al*., 1985; Westoby *et al*., 2002; Harrison *et al*., 2021). However, startup plants have not previously been compared to startup enterprises, to the best of our knowledge. Startups face the challenge of building productive capacity starting from limited initial revenue, and for seedlings especially, from limited capital reserves.

Differences between startups and mature plants arise through ontogenetic plasticity. There has been increasing interest in within‐species trait variation (e.g. Poorter *et al*., 2019; Westerband *et al*., 2021 and associated Special Issue), and plasticity is an important contributor to it. Often it may be better to think of traits as showing a characteristic trajectory through ontogeny, rather than a characteristic lifelong value.

We do not consider cotyledon‐stage seedlings, only true leaves. Some species have juvenile leaves that are morphologically distinct from adult foliage (heteroblasty; Zotz *et al*., 2011). However, quantitative trait shifts occur even when leaf shape and orientation are similar. It is not yet known whether heteroblastic species show stronger shifts in quantitative traits.

In this article, the evidence discussed is largely for LMA, because that is where most evidence is available and also where existing concepts (see later) make predictions. Other traits are discussed briefly in Supporting Information Notes S1. The broader message is that questions about differences between startups and established plants are ripe for study across a range of traits. Resprouts are different from seedlings in having larger reserves and an established root system, and in being in a microsite already proven able to support a plant. They are discussed in a separate section.

We consider three formulations for how regeneration strategy should be different, which for brevity we call startup firms, R‐shift, and trait‐growth theory. They make similar predictions on several points, but are not identical.

1. *Startup firms* are a recognized category in business. The analogy of startup plants to startup firms (Box 1) is, briefly, that they share a high failure rate, and a strong emphasis on generating positive cashflow as promptly as possible. Viability is assessed via the individual revenue‐producing unit (customer or leaf), notably through relating revenue to the cost incurred when acquiring or constructing the unit.
2. *R‐shift:* Dayrell *et al*. (2018) formulated the difference between startups and established plants as shifts within Grime’s (1977) CSR triangle from C (competition‐winning) or S (stress‐tolerant) toward R (ruderal). They attributed R‐shift to ‘the greater vulnerability of juveniles and the high costs of maintenance and reproduction in adults’. In their implementation R‐shift was measured by lower LMA. Low LMA confers high light‐capture area deployed per dry mass invested and is a strong predictor of rapid early seedling growth (Lambers & Poorter, 1992; Gibert *et al*., 2016). The R‐shift concept could be extended to other traits of ruderal species.
3. *Trait‐growth theory* (Gibert *et al*., 2016; Falster *et al*., 2018) shows how the influence of traits on growth rates changes with height or total leaf area of the plant (Box 2). For example, low LMA is a strong correlate of growth rates across species for seedlings, but this correlation disappears in larger plants (Gibert *et al*., 2016). As a corollary, trait values that maximize growth are expected to shift with height. In particular, the LMA that maximizes growth increases with plant height.

Box 2 **Trait‐growth theory in brief**

Two equations underpin trait‐growth theory (Falster *et al*., 2018). The first is the classic equation for growth of vegetative biomass d*B*/d*t*

(Eqn 1) $$\frac{dB}{dt}=\alpha \left(\frac{M_{l}}{\text{LMA}}\bar{p}\left(E\right)‐\sum_{i=l,b,s,r}M_{i}r_{i}\right)‐\sum_{i=l,b,s,r}M_{i}k_{i}$$

The right‐hand side of Eqn 1 can be thought of as three terms (the first two multiplied by α), which account in order for photosynthesis, respiration, and tissue turnover losses. The values of M,r and k refer to mass, maintenance respiration rate, and turnover rate for different tissues (leaf, bark and phloem, sapwood, and roots denoted by subscripts l, *b*, *s*, and *r*). Dividing by leaf mass per area (LMA) converts leaf mass *M*_l_ into leaf area, $\bar{p}\left(E\right)$ is the assimilation rate of CO_2_ per unit leaf area, and α adjusts gross primary production for growth respiration and other factors affecting the conversion of fixed carbon fixed into biomass.

The second key equation expresses the importance of allocation. For example, the rate of leaf area growth can be expressed as

(Eqn 2) $$\frac{dA_{1}}{dt}=\frac{dA_{1}}{dM_{a}}\cdot \frac{dM_{a}}{dB}\cdot \frac{dB}{dt}$$

The two terms on the right‐hand side in addition to d*B*/d*t* are

- $\frac{dA_{l}}{dM_{a}}$ the rate of deployment of new leaf area per mass invested, accounting for LMA and also for support costs elsewhere in the plant. Deployment is potentially increased by economizing on construction costs.
- $\frac{dM_{a}}{dB}$ the fraction of biomass produced that is allocated to vegetative growth vs to reproduction. Growth rate is increased by reducing investment in reproduction.

**Fig. 1:**
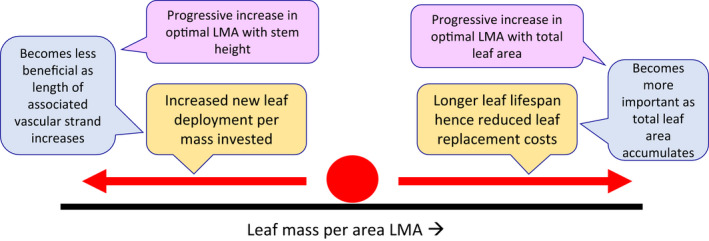
Schematic showing countervailing forces acting on the leaf dry mass per area (LMA) that maximizes growth rate, according to trait‐growth theory (Falster *et al*., 2018).

The leaf economic spectrum (Wright *et al*., 2004) expresses a trade‐off whereby species with lower LMA deploy more leaf area per leaf mass, but at the cost of shorter leaf lifespan. The plant’s height or total canopy area affects how LMA influences growth. In Eqn 1, lower LMA increases leaf deployment per mass in the first term, which is positive, but also increases turnover losses in the third term, which is negative. At the LMA that maximizes growth d*B*/d*t*, these two effects will be equal and opposite (Fig. 1). The negative effect on biomass production carries more weight as the total leaf area of the plant accumulates. From that mechanism, growth‐maximizing LMA should be higher in plants that have more total leaf area.

There is also an influence of costs associated with leaf construction, beyond the leaf itself. The taller the plant, the more vascular strand per unit leaf area, and this reduces the term $\frac{dA_{l}}{dM_{a}}$ in Eqn 2. Consequently, reducing LMA confers less benefit in lowering the total construction cost for leaf area than it would for shorter plants. This effect also, like the leaf turnover effect, shifts the growth‐maximizing LMA higher for larger plants (Fig. 1).

Basal resprouts and epicormic growth are not yet explicitly considered by trait‐growth theory, which in its current version has fixed allometries among foliage, stem and root.

## Leaf mass per area adjustment with height

**Fig. 2:**
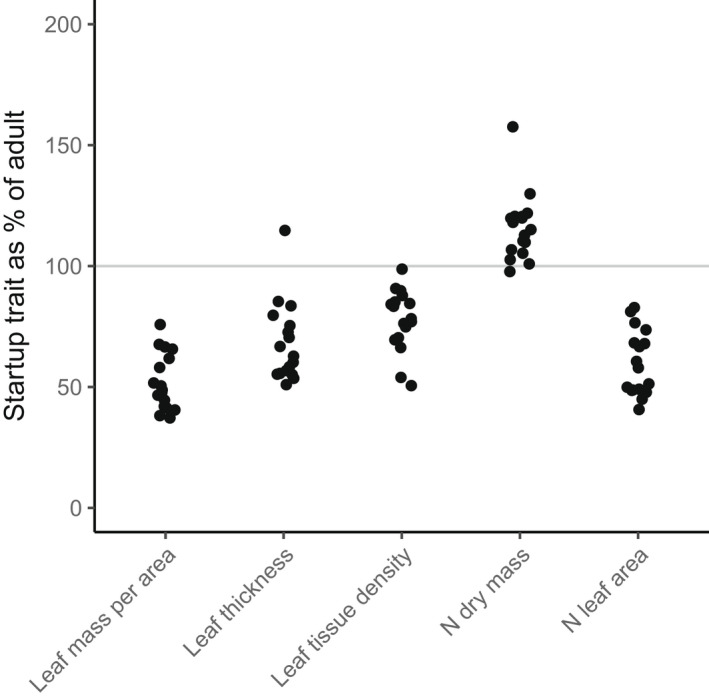
Traits of full‐light sapling leaves as a percentage of adult canopy leaves for 17 Guyana rainforest species (Houter & Pons, 2012). From left to right traits are leaf dry mass per area (LMA), leaf thickness, leaf tissue density, nitrogen (N) per leaf dry mass, N per leaf area.

All three framings of startup plant strategies – startup firms, R‐shift, and trait‐growth theory – predict that LMA will be lower in startup plants (Table 1, predictions 1–3). This appears to be almost universally true in the real world. For example, Houter & Pons (2012) compared leaves of full‐light juveniles (27 ± 13 cm tall) with upper canopies of adults across 17 tropical forest species. The sapling leaves averaged about half the LMA (Fig. 2), individual species ranging from *c.* 80% to 40%. Both leaf thickness and tissue density contributed to the difference (Fig. 2). Similarly, Thomas & Winner (2002) found that adult leaves had LMA 43% higher than full‐light saplings (0.5–2 m height) in *Pseudotsuga menziesii* and 80% higher in *Tsuga heterophylla*; and in a meta‐analysis of seven similar open‐canopy comparisons from the literature found a highly significant effect size of 2.5. Presumably some of the differences between species in quantity of LMA‐shift should be attributed to properties of the species or the situation, and some to experimental noise.

**Table 1:** Predictions or questions about traits of startup plants.

Prediction or question	Source or reasoning
(1) Lower leaf dry mass per area (LMA) in seedlings	Startup firm analogy: rapid return needed due to lack of reserves. R‐shift makes same prediction, also trait‐growth theory, see Box 2 for mechanisms.
(2) Progressive increase in LMA with height	Increasing non‐LMA costs of deploying unit leaf area reduce the benefit from lower LMA. Trait‐growth theory, see Box 2.
(3) Progressive increase in LMA with total leaf area	Increasing costs of replacing leaf turnover as leaf lifespan becomes shorter. Trait‐growth theory, see Box 2.
(4) Is increasing LMA with height associated with increasing leaf lifespan?	Longer leaf lifespan is required under trait‐growth theory for the advantage to accrue from reduced leaf replacement costs.
(5) No height effect on area‐basis photosynthetic capacity and total leaf nitrogen (provided no difference in light environment)	Assumed constant in trait‐growth theory; however, this is in the nature of a null assumption rather than a strong prediction. If true, mass‐basis photosynthetic capacity and leaf nitrogen are expected to decrease with height.
(6) Same LMA in basal resprouts as in seedlings or saplings, if matched for height	Prediction from trait‐growth theory on the basis that only height is an important influence.
(7) Lower LMA and lower stem density in multiple basal resprouts	Shorter lifetime or higher risk of mortality is acceptable, seeing that most of these resprouts will be discarded at an early stage. It is interesting also to ask whether lower stem tissue density is associated with shorter xylem lifespan in the same way as lower LMA is associated with shorter leaf lifespan.
(8) Higher LMA in basal resprouts than in seedlings	Startup firm analogy, less priority on rapid return since more reserves available than for seedlings.
(9) Lower LMA in basal resprouts than in seedlings	Aiming to restore balance between leaf area and roots as quickly as possible.
(10) LMA of epicormic growth increases with height	Predicted by trait‐growth theory if length of vascular strand is the main driving force.
(11) Lower LMA for epicormic regrowth following fairly complete defoliation as by crown fire, compared to following local defoliation	Predicted by trait‐growth theory if costs of replacing leaf are the main driving force.
(12) Are shifts in LMA or other traits more extreme where juvenile foliage has distinct morphology (heteroblastic)?	Perhaps distinct morphology is only selected for when particularly substantial changes are favoured in quantitative traits.
(13) Fast early growth (via low seedling LMA and possibly low stem tissue density) is favoured in stands where density‐dependent mortality is important	For example, at sites where seedlings often establish as crowded even‐aged stands, and there is substantial mortality before reproductive sizes are reached. Conversely, in situations where there is considerable threat of density‐independent mortality (drought, shade, herbivory), seedlings might be hypothesized to have more conservative traits. The hypothesis is that this difference in population dynamics accounts for some of the variation between species in the magnitude of trait difference between seedlings and adults. Generally, the consequences of mortality risk in regenerating plants can be argued two ways. One argument is that mortality risk selects strongly for rapid growth in order to gain height and escape from risks that apply particularly to small plants. Alternatively, allocation might be expected to avert particular risks – defence against herbivores, for example, or deeper roots – at the expense of faster growth. Mortality risk has yet to be integrated into trait‐growth theory.

Brief sketches of research needed to address each question are provided in Supporting Information Table S1.

In other comparisons within natural vegetation (e.g. Thomas & Bazzaz, 1999; Hölscher, 2004; Martínez‐Garza & Howe, 2005; Ishida *et al*., 2005; Kenzo *et al*., 2006, 2015; studies gathered in Poorter *et al*., 2009; Kitajima & Poorter, 2010; Liu *et al*., 2010; Spasojevic *et al*., 2014; Zhang & Wang, 2021; Martin & Isaac, 2021), seedling and sapling leaves have developed in shade compared to top‐of‐canopy leaves. These comparisons will have light‐response plasticity laid on top of effects of plant size, since LMA is known to decrease up to about three‐fold from high to low light intensities (Poorter *et al*., 2009). Similarly, a compilation by Cornelissen *et al*. (2003) comparing field‐grown adults with seedlings across 90 species showed median LMA *c.* 50% lower in seedlings, but the seedlings were from growth chambers at only 130 µmol m^–2^ s^–1^ photosynthetically active light for 14 h.

**Fig. 3:**
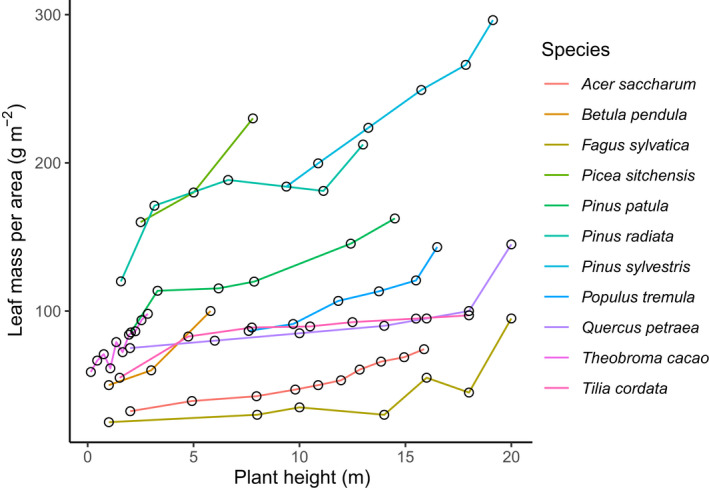
Leaf dry mass per area (LMA) in relation to height for several woody species. Re‐plotted from data compiled for Poorter *et al*. (2009), and kindly provided by him.

Another prediction is that the change in LMA will not just be a case of early seedling leaves being different, but rather will be progressive with height (prediction 2 in Table 1) or with total leaf area of the canopy (prediction 3 in Table 1). Fig. 3 shows that change in LMA is indeed progressive with height, as predicted. The prediction for progressive LMA‐shift is only made explicitly by trait‐growth theory. However, if R‐shift and startup firm framings were thought of as progressive change rather than as dichotomies, then arguably they also might predict progressive change in LMA.

There appear to be few measurements of leaf lifespan in association with the LMA shift with height comparing smaller with larger individuals (question 4 in Table 1). However, in two out of three *Helianthus* species (Mason *et al*., 2013), there was a distinct increase in LMA from juvenile to full vegetative growth, and this was indeed associated with increased leaf lifespan. The connection to leaf lifespan is relevant because one of the proposed selective forces favouring higher LMA in larger plants is reduced leaf replacement costs (Fig. 1; Box 2).

## Resprouts and epicormic growth

Resprouts and epicormic shoots are in a different situation from seedlings in several ways. They have an established root system in a setting where the species is proven to be able to grow successfully, they are often supported by more metabolic reserves than a seed provides, and usually there are numbers of shoots produced from a single genet. So then, what sort of trait differences should we expect?

Leaf mass per area of resprouting shoots is generally lower than for adult foliage, but often somewhat higher than for seedlings (e.g. Ishida *et al*., 2005; Peña‐Rojas *et al*., 2005; Salk, 2012). The simplest explanation for the higher LMA than seedlings might be that resprouts are generally larger and taller (prediction 6 in Table 1), and LMA is known to increase with height. Ramdial *et al*. (2020) indeed found that stump sprouts had similar LMA compared to saplings of the same size in three Surinam tree species. Therefore on the limited evidence available, it might be that height affects LMA in the same way for saplings and for stump sprouts.

Resprouts are often produced in numbers from many buds in a single genet, compared to the single shoot of seedlings. This has the obvious benefit of deploying leaf area faster, and rebalancing leaf : root ratio. However, these multiple shoots typically are sorted down to few or one quite quickly, as apical dominance is reasserted. Given that most of these shoots are not likely to persist, it might be predicted that they would be produced more cheaply than in seedlings, via lower LMA (prediction 7 in Table 1). Lower stem density might be expected on the same basis. Ramdial *et al*. (2020) indeed found that stump sprout stems were more slender and of lower density than sapling stems of the same height, with the result that stump sprouts were deploying more leaf area per unit wood mass.

Upper canopy epicormic sprouts may incur the costs of a longer conductive pathway, compared to lignotuber sprouts, assuming that new vasculature is produced rather than the new foliage being supported by existing vasculature. On the basis of trait‐growth theory, this should favour higher LMA, since gains in leaf lifespan are set against a smaller relative increase in total cost of the leaf (Box 2; prediction 10 in Table 1). Alternatively, if total leaf area and the cost of replacing leaf turnover were the dominant force, then epicormic shoots following complete canopy combustion should be similar to basal sprouts. Across 56 eucalypt species, juvenile foliage on resprouts did mostly have lower LMA than adult leaves (Vlasveld *et al*., 2018), but these data are not associated with records of the height from which juvenile foliage was collected.

## Future prospects

Possibilities for the future include theory development, topics not covered in this overview, and fresh experiments and field data especially.

The beginnings of quantitative theory for traits of startups exists. At the same time, there is much scope for developing it further and extending to a wider range of traits. Extension to resprouts and epicormic growth, integration of mortality risk, and better treatment of the consequences of varying stem tissue density are areas particularly deserving development.

Our coverage here has focused almost entirely on woody plants. Can similar questions and theoretical framings apply to herbaceous dicots and graminoids? Their foliage dies back at the end of the growth season, and this places a cap on the potential leaf lifespan benefit from increasing LMA. To some extent, the same complication applies to deciduous woody plants. The assumption in current trait‐growth theory of a fixed allometry does not apply well to herbs and graminoids. It is a question for the future whether the differences between startups and mature plants will be similar in herbs and graminoids compared to woody plants.

As Table 1 illustrates, many questions about traits of startup plants remain to be resolved. At the same time, a striking thing about the hypotheses or questions in Table 1 is that they are mostly quite susceptible to testing. We believe that trait ecology of startups is an area where rapid progress can be made both through developing theory and through empirical quantification.

## Competing interests

None declared.

## Author contributions

MW developed the concept in discussion with JS and DF, and wrote initial outline. JS and DF contributed further literature and theory, and critiqued successive drafts.

## Supporting information


**Notes S1** Leaf nutrients, water relations and defence.
**Table S1** The predictions or questions listed in main text Table 1, together with the field comparisons that would assess whether the prediction is correct.
Please note: Wiley Blackwell are not responsible for the content or functionality of any Supporting Information supplied by the authors. Any queries (other than missing material) should be directed to the *New Phytologist* Central Office.
