# Supplementary material for: Trait ecology of startup plants
Source: New Phytol. 2022 May 24;235(3):842–7. doi: 10.1111/nph.18193 (PMC9325420; doi:10.1111/nph.18193)
Supplement: Supplementary file 1 — Notes S1 Leaf nutrients, water relations and defence. Table S1 The predictions or questions listed in main text Table 1, together with the field comparisons that would assess whether the prediction is correct. Please note: Wiley Blackwell are not responsible for the content or functionality of any Supporting Information supplied by the authors. Any queries (other than missing material) should be directed to the New Phytologist Central Office. [file NPH-235-842-s001.pdf]

New Phytologist Supporting Information for “Trait ecology of startups”, Westoby M, Schrader J, Falster DS. Accepted 21 April 2022

Notes S1: Leaf nutrients, water relations and defence

Startup firm analogy, R-shift and trait-growth theory do not make clear predictions about leaf nutrients. It might be suggested that high leaf N is expected for seedlings in pursuit of rapid returns, but really this would depend on the cost of acquiring the N. For saplings in full light, Houter and Pons (2012) found leaf N per area substantially reduced compared to established plants, and leaf N per mass a little higher in association with lower LMA (Fig 2 in main text). Most available evidence on this question is confounded with seedling and sapling leaves developing in lower light than adult canopy foliage (e.g. Thomas & Bazzaz, 1999; Cornelissen et al., 2003; Hölscher, 2004; Ishida et al., 2005; Kenzo et al., 2006, 2015). Lower light is well established (Poorter et al., 2019) to produce decreases in LMA, total nitrogen per area and light-saturated photosynthetic rate among other traits.

For leaf water relations, the gravitational effect on water potential is only 0.01 MPa per m of height. Accordingly leaf level water relations are not expected to be strongly affected by height differences as such, in the range from ground level to say 10 m. (In trees reaching 100 m such as Koch et al’s (2004) *Sequoia sempervirens*, leaves at the top do have to operate at a full MPa more negative leaf water potential in order to draw up water.) A strong indirect effect of height on water relations might be expected inasmuch as shorter, younger plants will usually have shorter root systems and less access to deep soil water. How much this matters will depend, of course, on the climate and on wet and dry seasons. Seedling germination typically is organised such that initial growth occurs during a wet season, but surviving the first dry season is a challenge in many situations.

In a research garden of aspen (*Populus tremuloides*) genotypes followed over time (Cole et al., 2021), LMA decreased by about 20% from years 2 to 7, the only clear exception we know of to the generalization that LMA increases with height. In parallel, defensive phenolic glycosides and condensed tannins decreased by about 50%. Possibly this exceptional

behaviour might be accounted for by strong browsing impact from elk (*Cervus canadensis*) and other ground-feeding herbivores on regenerating aspen stands (Lastra et al., 2017).

A qualitative model proposed by Boege and Marquis (2005) suggested that allocation to defense is expected to increase from true-leaf seedling stage into sapling stage. Their argument was that as seedlings grow larger they have lower root-shoot ratios, more storage and more resources overall. In this argument defense is decided by resources available more so than by the value of defense; whereas in our view the question is about relative allocation within whatever resources are available. To us it appears there is no clear expectation based on resources available or on the relative importance of mortality at different sizes. Rather, differences in defense might be expected when the objective threat from herbivory changes with height, as in the example with aspen (Cole et al., 2021).

Table S1. The predictions or questions listed in main text Table 1, together with the field comparisons that would assess whether the prediction is correct

Prediction or question	Appropriate comparison
1. Lower LMA in seedlings	Seedlings vs adult canopy compared within species, with leaves developed under common light conditions. Leaves to be measured at different heights and on plants with different total leaf area. It would be valuable to obtain good estimates of within-species variation, in order to assess whether the change in LMA with height differs substantially between different species.
2. Progressive increase in LMA with height	
3. Progressive increase in LMA with total leaf area	
4. Is increasing LMA with height associated with increasing leaf lifespan?	Measure leaf lifespan in association with LMA at different heights on the plant.
5. No height effect on area-basis photosynthetic capacity and total leaf nitrogen (provided no difference in light environment)	Measure gas-exchange and nutrient traits of leaves in association with the changing LMA with height, on plants developing in common light environment.
6. Same LMA in basal resprouts as in seedlings or saplings, if matched for height	Compare LMA between basal resprouts and seedlings or saplings, across a range of heights so that they can be compared at common height.
7. Lower LMA and lower stem tissue density in multiple basal resprouts	For species that produce multiple basal resprouts, for example from lignotubers, compare stem tissue density and LMA with same-height seedlings or saplings.
8. Higher LMA in basal resprouts than in seedlings	For species that produce basal resprouts, compare stem tissue density and LMA with same-height seedlings or saplings.
9. Lower LMA in basal resprouts than in seedlings	
10. LMA of epicormic growth increases with height	Considering epicormic growth that extends along the height of a stem (for example after crown-consuming fire), compare LMA at different heights, using leaves at consistent length along the epicormic shoots.
11. Lower LMA for epicormic regrowth following fairly complete defoliation as by crown fire, compared to following local defoliation	Compare LMA for epicormic growth following complete defoliation with regrowth following local defoliation, controlling for height on the plant.
12. Are shifts in LMA or other traits more extreme where juvenile foliage has distinct morphology (heteroblastic)?	Compare the magnitude of the LMA shift with height between a sample of species with morphologically distinct juvenile foliage, and a sample without. LMA shift needs to be measured across a consistent height contrast. To the extent possible, distribute both samples widely across phylogeny, and construct species comparisons as phylogenetically independent contrasts.

13. Fast early growth (via low seedling LMA and possibly low stem tissue density) is favoured in stands where density-dependent mortality is important.	Construct two samples of species, one where seedlings typically establish as crowded even-aged stands, and there is substantial mortality before reproductive sizes are reached, and another where seedlings typically experience considerable density-independent mortality (drought, shade, herbivory). Compare magnitude of shift in LMA (and other traits) between these two groups of species. To the extent possible, distribute both samples widely across phylogeny, and construct species comparisons as phylogenetically independent contrasts.

References

Boege K, Marquis RJ. **2005**. Facing herbivory as you grow up: the ontogeny of resistance in plants. *Trends in Ecology & Evolution* **20**: 441–448.

Cole CT, Morrow CJ, Barker HL, Rubert-Nason KF, Riehl JFL, Köllner TG, Lackus ND, Lindroth RL. **2021**. Growing up aspen: ontogeny and trade-offs shape growth, defence and reproduction in a foundation species. *Annals of Botany* **127**: 505–517.

Cornelissen JHC, Cerabolini B, Castro-Díez P, Villar-Salvador P, Montserrat-Martí G, Puyravaud JP, Maestro M, Werger MJA, Aerts R. **2003**. Functional traits of woody plants: correspondence of species rankings between field adults and laboratory-grown seedlings? *Journal of Vegetation Science* **14**: 311–322.

Hölscher D. **2004**. Leaf traits and photosynthetic parameters of saplings and adult trees of co-existing species in a temperate broad-leaved forest. *Basic and Applied Ecology* **5**: 163–172.

Houter NC, Pons TL. **2012**. Ontogenetic changes in leaf traits of tropical rainforest trees differing in juvenile light requirement. *Oecologia* **169**: 33–45.

Ishida A, Yazaki K, Hoe AL. **2005**. Ontogenetic transition of leaf physiology and anatomy from seedlings to mature trees of a rain forest pioneer tree, Macaranga gigantea. *Tree Physiology* **25**: 513–522.

Kenzo T, Ichie T, Watanabe Y, Yoneda R, Ninomiya I, Koike T. **2006**. Changes in photosynthesis and leaf characteristics with tree height in five dipterocarp species in a tropical rain forest. *Tree Physiology* **26**: 865–873.

Kenzo T, Inoue Y, Yoshimura M, Yamashita M, Tanaka-Oda A, Ichie T. **2015**. Height-related changes in leaf photosynthetic traits in diverse Bornean tropical rain forest trees. *Oecologia* **177**: 191–202.

Koch GW, Sillett SC, Jennings GM, Davis SD. **2004**. The limits to tree height. *Nature* **428**: 851–854.

Lastra RA, Kenkel NC, Daayf F. **2017**. Phenolic Glycosides in Populus tremuloides and their Effects on Long-Term Ungulate Browsing. *Journal of Chemical Ecology* **43**: 1023–1030.

Poorter H, Niinemets Ü, Ntagkas N, Siebenkäs A, Mäenpää M, Matsubara S, Pons T. **2019**. A meta-analysis of plant responses to light intensity for 70 traits ranging from molecules to whole plant performance. *New Phytologist* **223**: 1073–1105.

Thomas SC, Bazzaz FA. **1999**. Asymptotic height as a predictor of photosynthetic characteristics in Malaysian rain forest trees. *Ecology* **80**: 1607–1622.
